# Effect of Iron Site Substitution on Magneto-Optical Properties of Bi-Substituted Garnets for Magnetic Hologram Memory

**DOI:** 10.3390/ma19010151

**Published:** 2026-01-01

**Authors:** Sumiko Bharti Singh Chauhan, Yuichi Nakamura, Shinichiro Mito, Lim Pang Boey

**Affiliations:** 1 Department of Electrical and Electronic Information Engineering, Toyohashi University of Technology, Toyohashi 441-8580, Japan; 2 Department of Electronic Engineering, National Institute of Technology, Tokyo College, Tokyo 193-0997, Japan

**Keywords:** Bi-substituted yttrium iron garnet, magneto-optical properties, metal-organic decomposition, figure of merit

## Abstract

We have developed a magnetic holographic memory using transparent bismuth-substituted rare-earth iron garnet as a next-generation optical memory. To realize this, a magnetic garnet with a large Faraday rotation angle and a moderately small extinction coefficient is required. In this study, we investigated the effect of Al or Ga substitution for the iron site of bismuth-substituted yttrium iron garnet (Bi/YIG) films on their magneto-optical properties. The Faraday rotation angle decreased with the amount of substitution, *x*, increase, for both Al- and Ga-substituted Bi/YIG, and a reversal of sign of rotation angle was only observed for Ga-substituted Bi/YIG, indicating a compensation composition. In the Al-substituted sample, due to small squareness, the residual Faraday rotation angle at zero magnetic field, |*θ*_R,res_|, gradually decreased above *x* = 0.5, whereas in the Ga-substituted sample, the squareness ratio increased with increasing substitution up to *x* = 2.0, and thus showed a peak at *x* = 1.5. The Curie temperature and extinction coefficient were reduced with increasing substitution amount. As a result of a decrease in extinction coefficient, *k*, the high figure of merit, (|*θ*_R,res_|/2π*k*) · λ was obtained around *x* = 1.5~1.9 for Ga and *x* = 2.1 for Al, while it was smaller than that of Bi/RIG we usually used.

## 1. Introduction

Recent information and communication technology advances have increased the amount of data to be processed. The conventional optical storage devices, such as CDs, DVDs, and Blu-ray disks, have almost reached their capacity limit; therefore, there is a demand for next-generation recording media to store such a large amount of data. A strong candidate for such media is a holographic memory that stores and reconstructs data using the principle of holography with light interference [1,2,3,4,5,6,7,8,9,10,11,12,13,14,15,16,17,18]. It offers the potential for high recording density by multiple recording and a fast data transfer rate by using two-dimensional signal patterns. Most of the research on holographic memory uses photopolymers as recording media [19,20], whereas these materials are not rewritable and have issues with long-term stability. To overcome these issues, we have developed magnetic holographic memory using a transparent and stable magnetic garnet as a recording material [21,22,23,24,25,26,27,28,29]. For magnetic holographic memory, the data are recorded as the direction of magnetization corresponding to interference patterns. The thermomagnetic recording method is used for recording a magnetic hologram, and the magneto-optical (MO) effect, such as the Faraday effect, is used for reconstruction of the magnetic hologram [18,30,31,32]. To achieve bright reconstruction images of magnetic holograms, a large Faraday rotation angle is necessary, which can be obtained by the formation of deep magnetic fringes on a recording material with a large Faraday rotation angle, so the recording materials with a large rotation angle and an appropriately small extinction coefficient are required.

Magnetic garnets are a good candidate for such a recording material [33,34], and their magnetic and optical properties can be changed by appropriate elemental substitution [35,36,37,38,39,40,41,42,43,44,45,46,47,48,49,50,51,52]. Among the various types of garnets, Bi/YIGs have attracted much attention due to their large Faraday rotation angle and high optical transmittance in the visible and near-infrared range. Robertson et al. [43] reported that the addition of Bi YIG increased the Faraday rotation by ten times in comparison to pure YIG. Ishibashi et al. [45] investigated the effect of Bi-substituted YIG films prepared by the metal–organic decomposition (MOD) method and found that the Faraday rotation angle of the sample with Bi contents of *x* = 2.5 in Y_3−*x*_Bi*_x_*Fe_5_O_12_ reached the value of 1.55 × 10^5^ degree/cm at the wavelength of 524 nm.

In addition to their well-known role in enhancing magneto-optical effects, bismuth-containing oxide materials have also attracted attention in a broader context as multifunctional systems in which magnetic, electric, and optical responses can coexist or couple. For example, multiferroic behavior and magneto–electric–optical coupling have been reported in Bi-based oxide films such as BiFeO_3_-derived systems, demonstrating the versatility of bismuth incorporation in functional magnetic oxides [53,54]. Although the Bi-substituted iron garnet films investigated in this work are not ferroelectric and are primarily optimized for magneto-optical performance, these related developments highlight the broader functional opportunities offered by Bi-containing oxide materials and motivate continued exploration of Bi-based garnets for advanced opto-magnetic device applications, including magnetic holographic memory.

Motlagh et al. [46] investigated the effects of Al substitution for the iron site in the YIG using the mechanochemical method and reported that the Al element initially preferred to occupy the tetrahedral sites while also occupying the octahedral sites as the amount of Al increased. In addition, the Al substitution led to an increase in coercivity but also to a decrease in the Curie temperature [46,49]. Zaini [50] reported that the Ga substitution in YIG decreased saturation magnetization and increased coercivity due to weak super-exchange interactions and grain growth. Similarly, Hamasha et al. [51] showed Ga substitution in YIG causes a decrease in saturation magnetization by replacing Fe ions with Ga ions at tetrahedral sites, which causes a decrease in lattice constants. Sasaki et al. [52] investigated the properties and magneto-optical behavior of Nd_0.5_Bi_2.5_Fe_5−y_Ga_y_O_12_ thin film and reported that the substitution of Ga increased the Faraday rotation angles and the transmittance.

As previously mentioned, the recording materials for magnetic holographic memory require not only a large Faraday rotation angle and an appropriately small extinction coefficient, but also good squareness, which means a large residual Faraday rotation in a zero magnetic field angle and a large coercivity. Good squareness in hysteresis loop is particularly important because it directly determines the residual Faraday rotation available for hologram recording and reconstruction. Therefore, in this work, the effects of Al or Ga substitution on the iron site of the Bi/YIG on the magneto-optical properties were investigated to find materials suitable for the magnetic hologram memory. The Bi/YIG samples with various Al or Ga contents were prepared with the MOD method, and their magneto-optical and optical properties were evaluated. In this study, magneto-optical measurements are used as practical indicators of magnetic performance, since the residual Faraday rotation reflects the combined influence of magnetization magnitude and hysteresis squareness.

## 2. Materials and Methods Experimental

The MOD method was used to fabricate the samples on Eagle XG glass substrate of size 12 mm × 12 mm. The MOD solutions were prepared by mixing solutions manufactured by Kojundo Chemical Laboratory with the cation ratios of Bi/Y/Al/Fe = 1.5:1.5:x:(5.0 − x) (x = 0.5~2.3) (hereafter (Bi,Al)/YIG) and Bi/Y/Ga/Fe = 1.5:1.5:x:(5.0 − x) (x = 0.5~2.3) (hereafter (Bi,Ga)/YIG). The MOD process consists of four steps: spin-coating, drying, calcination, and sintering. A cleaned glass substrate was placed on a spin coater, and the solution was deposited at 3000 rpm for 60 s. The coated substrate was then dried on a hot plate at 100 °C for 10 min and then calcined at 450 °C for 10 min. The calcination temperature of 450 °C was selected to effectively remove organic constituents from the MOD precursor after each coating step; this temperature is commonly used in MOD-based garnet processing and is sufficient to decompose metal–organic compounds while forming an amorphous oxide network without initiating crystallization [45]. During the iterative coating and calcination process, the films remained visually uniform after each cycle, indicating stable film formation. The repeated calcination steps ensured effective removal of residual organic components. This coating to calcination process was repeated five times to increase the film thickness. The calcined samples were sintered at 700 °C for 3 h for crystallization. The sintering temperature of 700 °C was chosen to crystallize the Bi/YIG garnet phase while minimizing undesirable effects such as bismuth volatilization and substrate deformation, since previous studies have reported that Bi-substituted YIG garnets crystallize in the range of 600–850 °C, depending on processing conditions [39,45]. After the final sintering step, the films were transparent and free of visible discoloration, suggesting complete burnout of organic binders. Although minor non-uniformity of thickness across the 12 mm × 12 mm substrates cannot be completely excluded, no macroscopic inhomogeneity was observed. This process from coating to sintering was repeated three times to obtain a total of 15 coating films to complete the samples.

The prepared samples were investigated by X-ray diffraction (XRD) using an X-ray diffractometer (RINT-2500, Rigaku Co., Tokyo, Japan) to identify the crystalline phase, and their optical and magneto-optical properties, such as transmittance spectra and Faraday rotation angles, were measured. No significant difference in surface morphology was observed under optical microscopy. For all the samples, the film thickness and the optical parameters, such as extinction coefficient, were evaluated by fitting the optical transmittance and absorbance spectra using SCOUT software (version 5.3; available at https://www.wtheiss.com).

## 3. Results and Discussion

### 3.1. XRD Results

**Figure 1:**
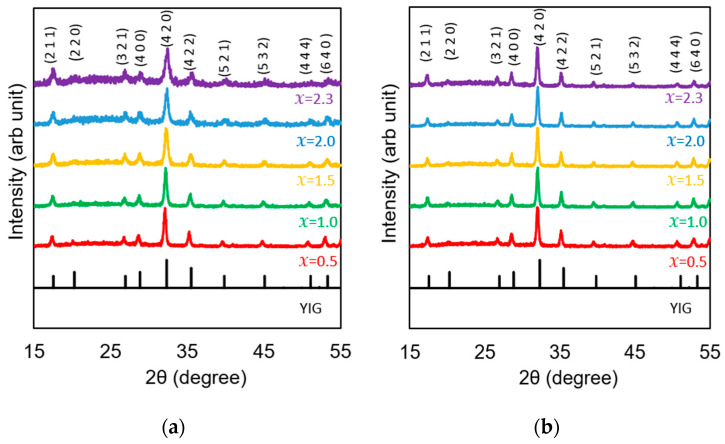
The XRD results of (**a**) (Bi,Al)/YIG and (**b**) (Bi,Ga)/YIG films.

The phases of the samples were identified using X-ray diffraction. The XRD patterns of the (Bi,Al)/YIG and (Bi,Ga)/YIG samples are shown in Figure 1a and Figure 1b, respectively. According to these results, all samples showed similar 2θ values and peak intensity patterns to YIG and were thought to be crystallized as garnet, and no peak from another phase was observed. So, the garnet was the major phase in these films, although the existence of a small amount of residual amorphous or secondary phases below the XRD detection limit cannot be excluded.

### 3.2. Lattice Constant

**Figure 2:**
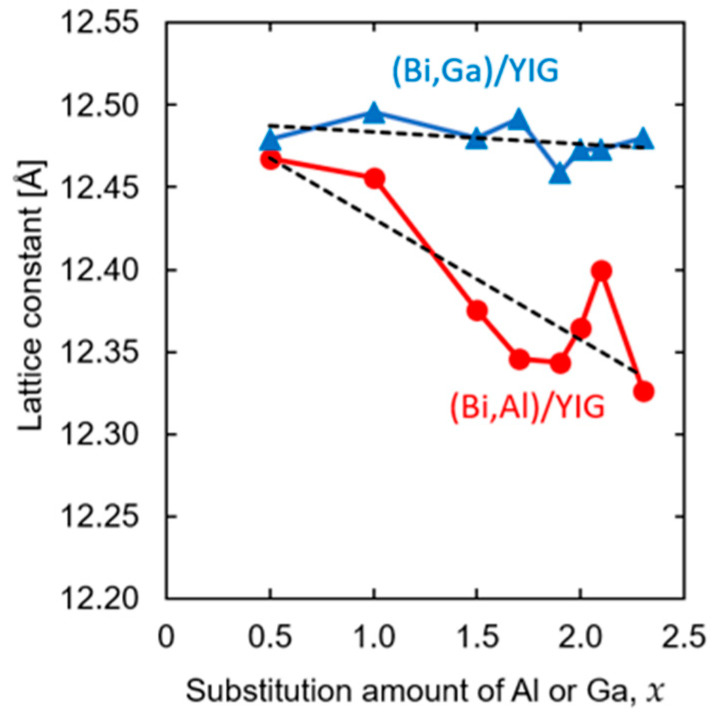
Lattice constant for (Bi,Al)/YIG and (Bi,Ga)/YIG films. The dashed lines represent linear fitting trends.

Lattice constants of (Bi,Al)/YIG and (Bi,Ga)/YIG films were evaluated from the XRD results using the least-squares method. The evaluated lattice constants are shown in Figure 2. The lattice constant of Al-substituted bismuth–iron–garnet ((Bi,Al)/YIG) decreased as the substitution amount of Al increased, while that of (Bi,Ga)/YIG remained almost constant. This is attributed to the difference in ionic radius; the ionic radius of Al^3+^ is 0.535 Å, which is smaller than that of Fe^3+^ (0.645 Å), while the ionic Ga^3+^ (0.62 Å) is closer to that of Fe^3+^, causing minimal structural distortion. This tendency is the same as the previously reported ones, and the change in the ionic radius results in the change in lattice constant through ionic distances, which may affect the properties of garnets [48,55,56].

### 3.3. Magneto-Optical Properties

**Figure 3:**
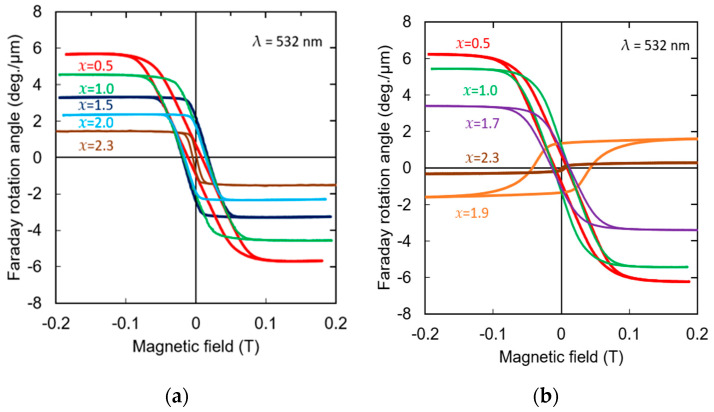
Faraday loops measured at the wavelength of λ = 532 nm for (**a**) (Bi,Al)/YIG and (**b**) (Bi,Ga)/YIG films.

The Faraday rotation measurements were performed at a wavelength of 532 nm. Figure 3a and Figure 3b show Faraday loops of the (Bi,Al)/YIG and (Bi,Ga)/YIG samples at room temperature, respectively. The loop area changed with the amount of Al and Ga substitution, and the absolute value of the Faraday rotation angle decreased as the amount of substitution for both Al- and Ga-substituted samples increased. The sign of the rotation angle was negative for all Al-substituted samples, while that of Ga-substituted samples changed from negative to positive around *x* = 1.7 to *x* = 1.9, which means the compensation composition existed around the composition.

This sign reversal of Faraday rotation may be attributed to the magnetic compensation behavior of ferrimagnetic garnets. In such systems, the Faraday rotation is governed by the dominant Fe^3+^ sub-lattice magnetization. When Ga^3+^ replaces Fe^3+^ ions at tetrahedral sites, this substitution may change the balance between tetrahedral and octahedral sub-lattice contributions and reduce the resultant ferrimagnetic moment. As a result, a compensation condition can be reached where the magneto-optically active Fe sub-lattice reverses its orientation with respect to the applied field, leading to a sign change in the Faraday rotation [57]. In addition, Faraday rotation is governed by wavelength-dependent electronic transitions and can be modified by Ga occupation through changes in the Fe-related electronic structure. Since the present measurements were performed at a single wavelength (532 nm), the relative contributions of magnetic compensation and electronic transition effects cannot be fully separated. Hence, a Faraday rotation spectra analysis is required for a more rigorous interpretation of the band-structure-related origin of the sign reversal in future.

On the other hand, the coercivity of the samples increased as the substitution amount increased up to *x* = 1.5 for Al and up to *x* = 1.9 for Ga-substituted samples, and then decreased.

**Figure 4:**
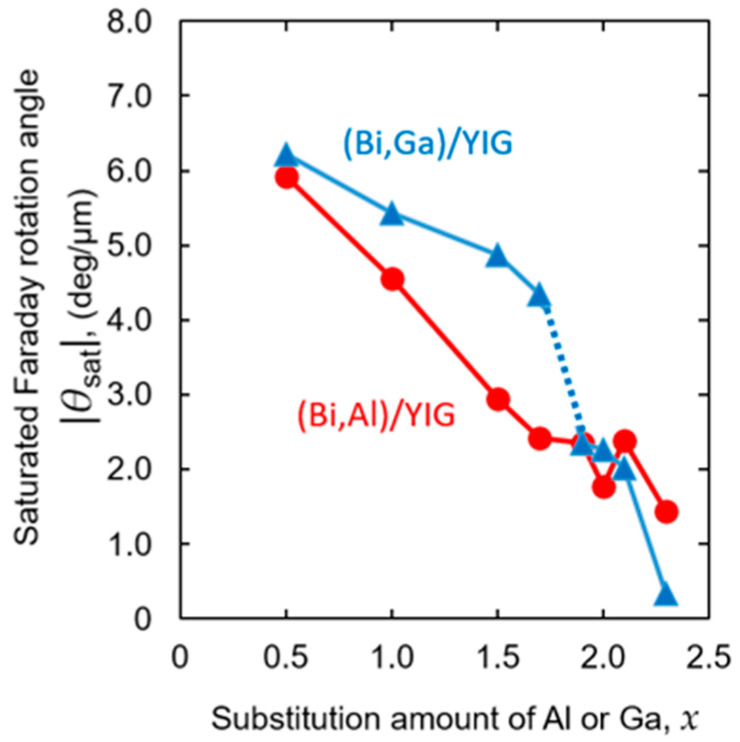
Composition dependence of the absolute value of the saturated Faraday rotation angle for (Bi,Al)/YIG and (Bi,Ga)/YIG films at the wavelength of *λ* = 532 nm. The dashed line is a guide to the eye.

Figure 4 shows the compositional dependence of the absolute values of the Faraday rotation of both Al- and Ga-substituted samples. As shown in Figure 4, the Faraday rotation decreased with increasing the substitution amount, as mentioned previously. Still, the manner of decrease was different between the Al-substituted and Ga-substituted samples. The Al-substituted sample showed a relatively linear decrease, whereas the Ga-substituted sample showed a slow decrease up to about *x* = 1.7, after which the rotation angle decreased rapidly. This reduction in the Faraday rotation angle would be attributed to the reduction in magnetic Fe^3+^ ions by non-magnetic ion substitution. The difference in the decrease in the rotation angle versus the amount of substitution between Al and Ga indicates that the substitution sites of Al and Ga may be different. There are two Fe sites in magnetic garnet; that is, three tetrahedral sites and two octahedral sites, and the magnetic properties change depending on how each site is substituted with nonmagnetic elements such as Al and Ga. As mentioned previously, the sign of the Faraday rotation angle of (Bi,Ga)/YIG changed between *x* = 1.7 and 1.9, which may be a reason for the change in the reduction rate of the rotation angle. The substitution of a nonmagnetic ion changed the local cation distribution, affecting the overall magnetic moments, especially due to this difference in the Ga substitution site, changing the sign of the rotation angle [57,58].

**Figure 5:**
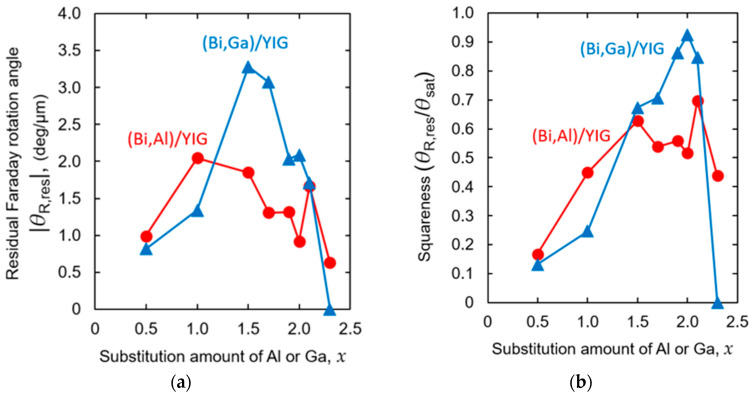
Composition dependence of the absolute value of (**a**) the residual Faraday rotation angle and (**b**) the squareness of (Bi,Al)/YIG and (Bi,Ga)/YIG at the wavelength of *λ* = 532 nm.

Figure 5a,b show the absolute value of residual Faraday rotation angles at zero magnetic field, which is important for the usage without a magnetic field, such as magnetic holographic memory, and the squareness of the Al- or Ga-substituted samples, respectively. For both Al and Ga substitution, the squareness of the Faraday loop is poor at low substitution amounts, as shown in Figure 5b, and the residual Faraday rotation angle gradually increases with the improvement of squareness. Then, the residual rotation angle of the Al-substituted samples peaked around *x* = 1.5 and gradually decreased, whereas the Ga-substituted samples peaked around *x* = 1.5 and decreased sharply near *x* = 2.0. At higher substitution amounts, although the squareness of the Ga-substituted samples varies, the squareness is basically as high as about 0.7, so the decrease in the residual rotation angle was due to the decrease in the saturation rotation angle caused by the substitution of Fe with nonmagnetic Al and Ga, as shown in Figure 4.

**Figure 6:**
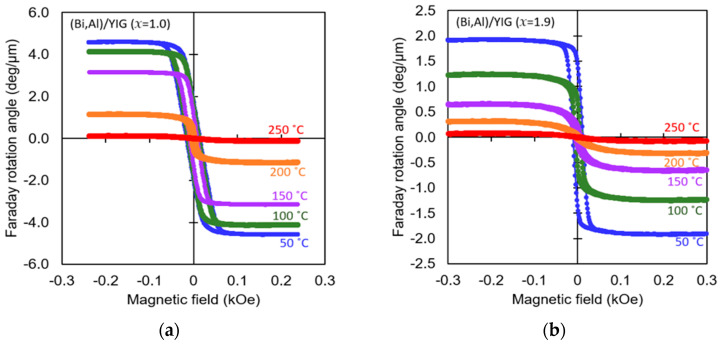

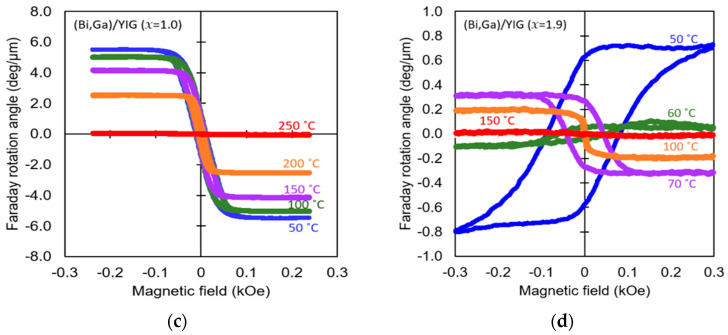
Faraday loops at various temperatures for (Bi,Al)/YIG with (**a**) *x* = 1.0 and (**b**) *x* = 1.9, and for (Bi,Ga)/YIG with (**c**) *x* = 1.0 and (**d**) *x* = 1.9, measured at a wavelength of λ = 532 nm.

Figure 6 shows the Faraday loops at various temperatures for (Bi,Al)/YIG and (Bi,Ga)/YIG samples. As shown in this figure, except for the sample with Ga substitution of *x* = 1.9, the absolute value of the Faraday rotation angle decreased monotonically with increasing temperature. In contrast, in the sample with Ga substitution of *x* = 1.9, the absolute value of the Faraday rotation angle first decreased rapidly, and then the sign of the Faraday rotation angle changed from positive to negative when the temperature increased from 60 °C to 70 °C, after that, the Faraday rotation angle decreased monotonically. This means the (Bi,Ga)/YIG sample with *x* = 1.9 has the compensation temperature between 60 °C and 70 °C. This is consistent with the existence of compensation composition around *x* = 1.7 and 1.9. The Fe site of magnetic garnet has three tetrahedral sites and two octahedral sites, and the magnetic properties change depending on how each is substituted with nonmagnetic elements such as Al and Ga. In this study, the sign of the Faraday rotation angle was always negative for the Al-substituted samples, but the compensation composition and compensation temperature were observed in the Ga-substituted samples, where the sign of the rotation angle changed depending on the composition and temperature. This suggests that the site occupancy of Al or Ga in garnet is different. Mohaidat et al. [48] reported that Al is substituted at the octahedral site, but, as the amount of Al increases, it starts to occupy the tetrahedral site [59], whereas Gilleo and Geller [55] reported that Ga can be substituted at the tetrahedral site. For Ga-substituted samples around *x* = 1.0, approximately 0.9 formula units occupy the tetrahedral site and 0.1 formula unit occupies the octahedral site [60]. This would be consistent with the fact that the substitution of Ga at the tetrahedral site weakens the ferrimagnetism of magnetic garnets and causes the appearance of a compensation composition.

**Figure 7:**
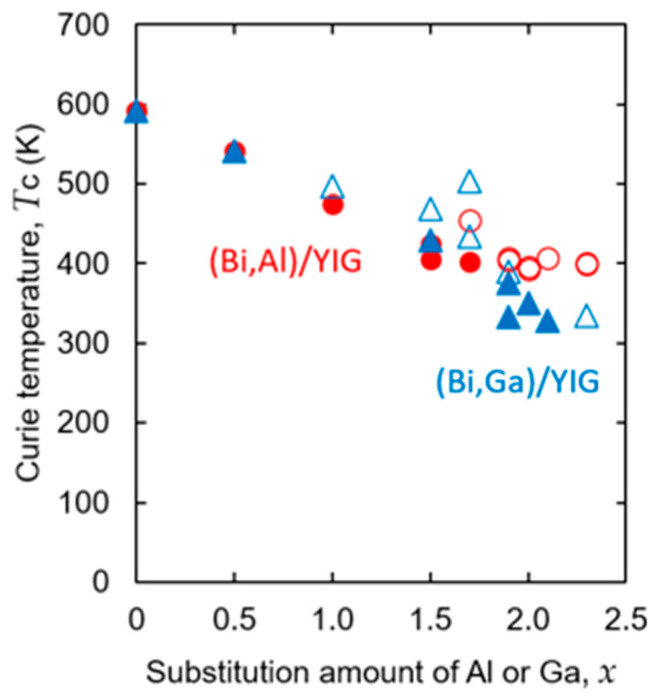
Composition dependence of the Curie temperature (*T*_C_) for (Bi,Al)/YIG and (Bi,Ga)/YIG films. Filled symbols indicate that the occupancy ratio *z*/*x* could be calculated, while open symbols indicate that the *z*/*x* ratio could not be calculated.

From the temperature dependence of these Faraday rotation angles, the Curie temperatures, *T*_C_, were evaluated by extrapolating to the temperature at which the rotation angle becomes zero [55], and the evaluated Curie temperatures are summarized in Table 1 and Figure 7 for (Bi,Al)/YIG and (Bi,Ga)/YIG samples. In both Al- and Ga-substituted samples, the Curie temperature decreases with the substitution of Al or Ga increases due to the replacement of magnetic Fe^3+^ ions by non-magnetic Al^3+^ or Ga^3+^. Both Al and Ga-substituted samples showed a similar decreasing trend.

**Figure 8:**
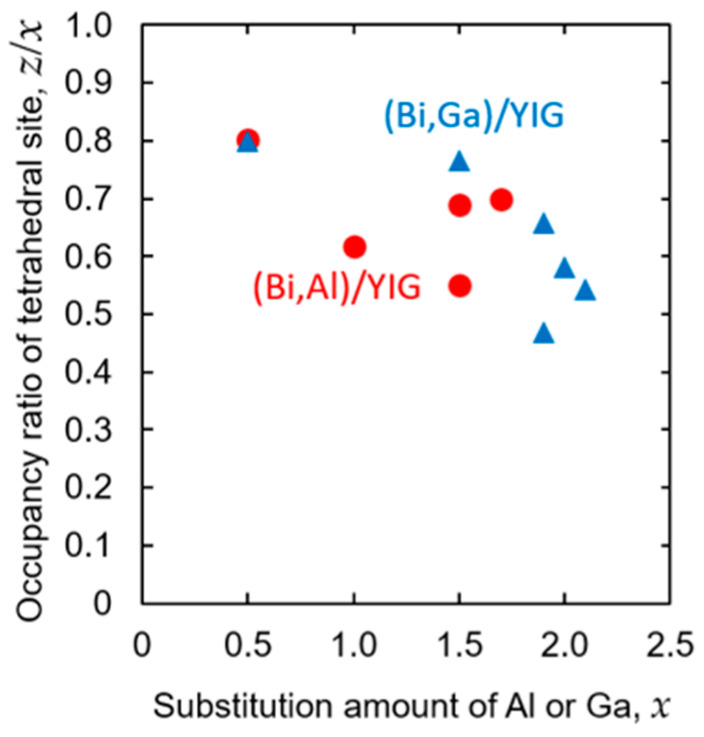
The evaluated tetrahedral sites occupancy ratio (*z/x*) as a function of substitution amount for (Bi,Al)/YIG and (Bi,Ga)/YIG films.

Based on these evaluated Curie temperatures, the occupancy ratio of the tetrahedral site, *z*/*x*, was evaluated using the method of Gilleo et al. [55,61] and summarized in Table 1, although the occupancy ratio could not be determined for some samples shown as “–” in Table 1. The Curie temperatures of such samples are shown as open symbols in Figure 7. Such samples are found in samples with a large amount of substitution with a low Curie temperature and showed relatively high Curie temperatures among samples with similar amounts of substitution. Actually, the temperature properties of the rotation angle tended to show a tail that may be due to minute impurities when the rotation angle becomes smaller in highly substituted samples, so it is thought to result in evaluating the Curie temperature higher than the actual value during extrapolation, combined with the low Curie temperature. Although it was not possible to evaluate the substitution ratio for some samples, the evaluated occupancy ratio of the tetrahedral site is shown in Figure 8 against the substitution amount. It should be emphasized that the occupancy ratio (*z*/*x*) was evaluated from the Curie temperature data using the Gilleo–Geller model and not determined by direct structural analysis. This model assumes an ideal cation distribution, so the evaluated *z*/*x* ratios should be regarded as qualitative trends rather than definitive evidence of Al or Ga site occupancy. A precise determination of substitution sites would require direct structural probes such as neutron diffraction.

As shown in Figure 8, Al and Ga ions primarily occupy the tetrahedral Fe sites at lower substitution levels (*x* ≤ 1.7), resulting in *z*/*x* values about 0.7–0.8. At low substitution amount (*x* = 0.5), Al ions and Ga ions primarily occupy tetrahedral Fe sites with a *z*/*x* value of approximately 0.8. As the substitution amount increases, the occupancy ratio of the tetrahedral sites tends to decrease to approximately 0.5–0.6 at a substitution amount of *x* ≥ 1.9. When comparing Al and Ga, the occupancy ratio of the tetrahedral sites of Ga was found to be higher at *x* = 1.5. However, since we could not compare at other substitution amounts, no clear tendency was observed regarding the differences in occupancy ratio by the substitution element. As shown in Figure 7 and Figure 8, some compositions exhibited different Curie temperature values despite having the same nominal substitution level. These variations may be attributed to local inhomogeneity of garnet composition. In addition, at high substitution levels, the Faraday rotation became small, so the effect of residual amorphous or secondary phases became large, which increases the uncertainty in extrapolating the Curie temperature. These factors collectively contribute to the observed spread in Curie temperature values.

**Figure 9:**
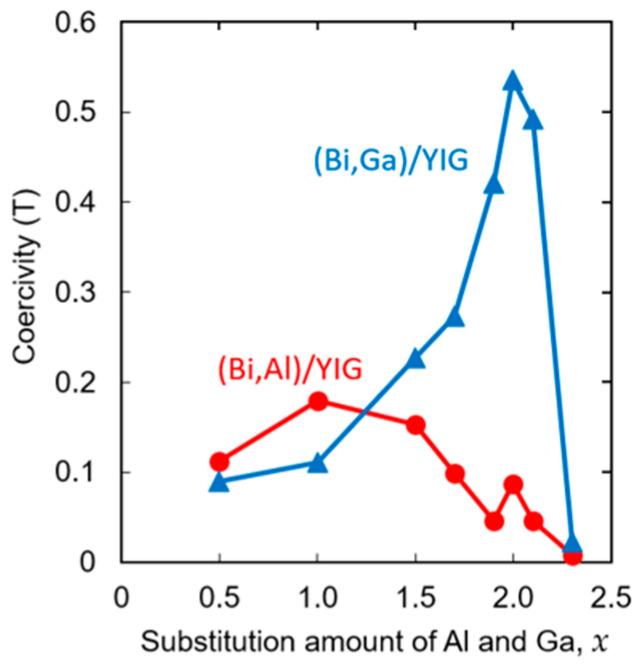
Composition dependence of coercivity for (Bi,Al)/YIG and (Bi,Ga)/YIG films.

Figure 9 shows the composition dependence of coercivity of (Bi,Al)/YIG and (Bi,Ga)/YIG. For (Bi,Al)/YIG, the coercivity increases moderately up to *x* = 1 and then decreases. In contrast, (Bi,Ga)/YIG exhibits a sharp increase in coercivity, peaking at *x* = 2.0 with the coercivity value exceeding 0.5 T, followed by a rapid decline to *x* = 2.3. This large coercivity may be related to the compensation composition close to this composition.

The observed composition dependence of coercivity may be qualitatively understood in terms of magnetic domain wall pinning in garnet thin films. In general, coercivity in ferrimagnetic garnets is governed by domain wall motion. Moderate Al or Ga substitution may introduce lattice distortion and internal stress, which can enhance domain wall pinning and lead to an increase in coercivity. This effect may be strong in the Al-substituted samples rather than the Ga-substituted samples because the change in lattice constant of the Al substitution sample was larger than that of Ga-substituted samples, as shown in Figure 2. However, the exceptionally large coercivity observed near *x* ≈ 2.0 of the Ga-substituted samples cannot be explained by this mechanism but can be associated with the proximity to a magnetic compensation composition, where the reduced net magnetization makes magnetization reversal energetically unfavorable [62].

### 3.4. Transmittance Properties

**Figure 10:**
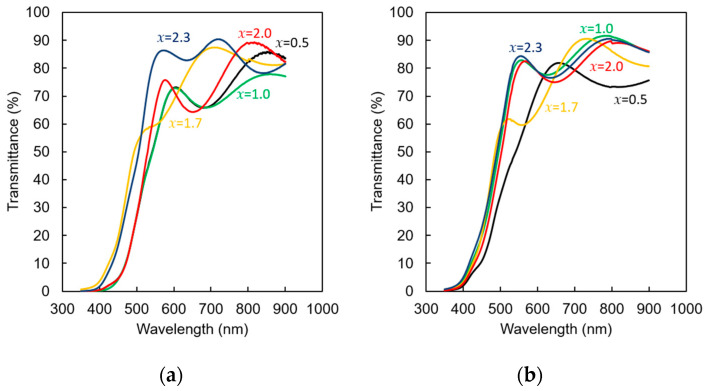
Transmittance spectra of (**a**) (Bi,Al)/YIG and (**b**) (Bi,Ga)/YIG films.

Figure 10 shows the results of transmittance spectra for (a) (Bi,Al)/YIG and (b) (Bi,Ga)/YIG, respectively. We can see that all samples show relatively high transmittance of 80% above 500 nm, but a sharp drop in transmittance occurs around 400–500 nm. This strong absorption below 400 nm is due to charge transfer transitions from the oxygen 2*p* orbital to the iron 3*d* orbital. In both the (Bi,Al)/YIG and (Bi,Ga)/YIG samples, an increase in the substitution amount increases the transmittance in the 500–800 nm range. Meanwhile, (Bi,Al)/YIG samples with *x* = 2.3 showed higher transmittance in the 400–500 nm range. (Bi,Ga)/YIG samples with *x* ≥ 1.0 showed a higher transmittance in the same range compared to the lower amount of substitution.

**Figure 11:**
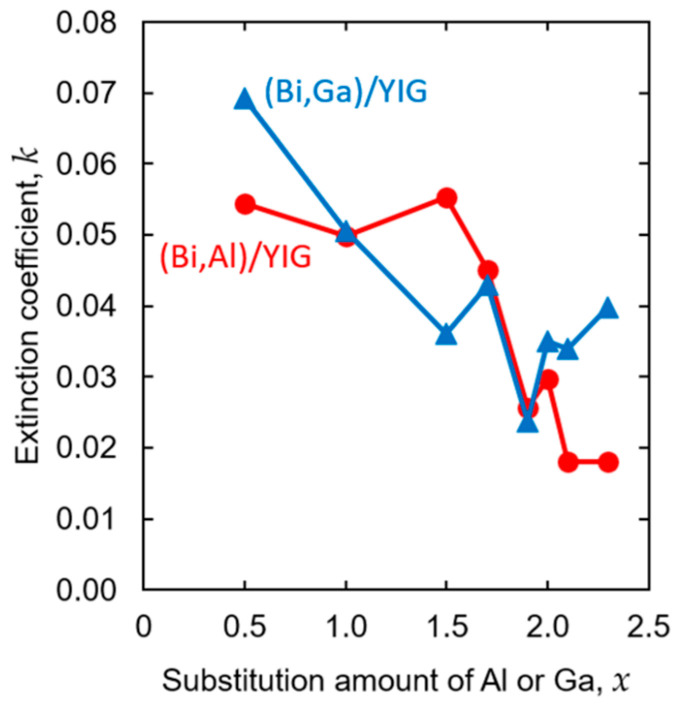
Composition dependence of the extinction coefficient, *k*, for (Bi,Al)/YIG and (Bi,Ga)/YIG films at the wavelength of *λ* = 532 nm.

From these transmittance spectra, the extinction coefficients were evaluated by fitting transmittance and absorption spectra using Scout software. The evaluated results are summarized in Figure 11. In both Al- and Ga-substituted samples, the extinction coefficient tended to decrease with increasing substitution amount. In the Ga-substituted samples, the extinction coefficients tended to decrease monotonically up to *x* = 1.9 with small fluctuation and showed a variation around *k* = 0.03–0.04 for higher substitution levels *x* = 2.1–2.3. Whereas, in the Al-substituted samples, the extinction coefficient remains almost similar between *x* = 0.5 and *x* = 1.5, but shows a noticeable decrease for higher substitution levels of *x* ≥ 1.9. This decrease in the extinction coefficient means that the Fe site is replaced by a nonmagnetic ion, which weakens the interaction between the Fe ion and light, resulting in a decrease in the rotation angle and also weakening the light absorption. This difference in behavior with substitution elements of Al and Ga is thought to be due to the difference in the substitution sites and in the electronic structure, but the details are not clear at the present stage.

From the results of the extinction coefficient, *k*, and the residual Faraday rotation angle, *θ*_R,res_, the figure of merit is defined as

(1) $$FOM=\frac{\left|\theta_{R,res}\right|}{2\pi k}\;\lambda$$

Here, *θ*_R,res_ represents the residual Faraday rotation angle at zero magnetic field after magnetization. This definition is appropriate for magnetic holographic memory, because the readout signal is determined by the residual Faraday rotation in the absence of an external magnetic field. As a result, the figure of merit depends not only on the conventional saturated Faraday rotation angle and optical absorption, but also on the magnetic squareness of the film.

**Figure 12:**
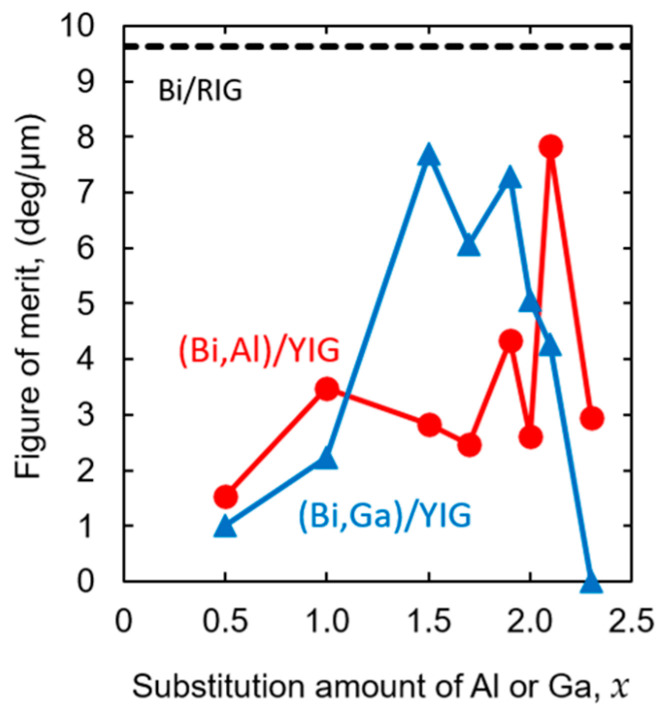
Figure of merit for (Bi,Al)/YIG and (Bi,Ga)/YIG films.

For non-magnetic field applications, the results were calculated, and the data are presented in Figure 12 for (Bi,Al)/YIG and (Bi,Ga)/YIG. The broken line shows the FOM value of Bi/RIG that we generally use. For the (Bi,Al)/YIG samples, the FOM initially increased and then remained almost constant except for a peak at *x* = 2.1. On the other hand, in the (Bi,Ga)/YIG samples, the FOM values initially increased with increasing the amount of Ga, corresponding to the decrease in the extinction coefficient, and showed a peak around *x* = 1.5–1.9, then rapidly decreased due to a rapid decrease in Faraday rotation angle and the high extinction coefficient of (Bi,Ga)/YIG.

Among the samples fabricated this time, high FOM values were obtained for the (Bi,Ga)/YIG with Ga contents of around *x* = 1.5 to 1.9, except for the momentary peak of the Al-substituted sample at *x* = 2.1. However, those values were still lower than those of the Bi/RIG sample we usually used. This is due to the poor squareness of the samples fabricated this time and the large extinction coefficient, so the improvements of those properties are required.

## 4. Conclusions

To find a suitable composition for the recording medium of magnetic holographic memory, Bi/YIG films with Al and Ga substitution at the Fe site were fabricated by the MOD method, and their magneto-optical properties were investigated. Both Al- and Ga-substituted samples showed a decrease in Faraday rotation angle as the amount of substitution increased, while the sign of rotation angle was reversed only in (Bi,Ga)/YIG around *x* = 1.5–1.7, suggesting a magnetic compensation composition. The residual Faraday rotation angle in the zero magnetic field showed relatively high values at substitution amounts of *x* = 1.0 to 1.7 due to poor squareness of the hysteresis loop at low substitution amounts, but the values were not large. Coercivity increased with substitution up to *x* = 1.5 for Al and *x* = 1.9 for Ga, and decreased at higher amounts of substitution. The Curie temperature decreased with increasing substitution, and the site occupancy analysis suggested that tetrahedral Fe sites were preferentially occupied at low substitution levels, but no clear differences were identified between Al and Ga substitution. With Fe site substitution, transmittance became large, and extinction coefficients decreased. As a result, the highest figure of merit was achieved at *x* = 2.1 for Al and *x* = 1.5–1.7 for Ga but did not exceed that of Bi/RIG we usually used due to low residual rotation angle and high extinction coefficient to achieve higher figure of merits, it would be effective that the substitution of other elements such as Dy, which increases magnetostriction, to improve the squareness of the Faraday loop, and to slightly reduce the amount of Bi substitution to lower the extinction coefficient.

In addition, maintaining a sufficiently high Curie temperature is essential for ensuring thermal stability in practical magnetic holographic memory applications. Therefore, future optimization must balance improvements in hysteresis loop squareness and optical loss reduction with the requirement of adequate Curie temperature, which will be the focus of subsequent studies. Furthermore, direct magnetic measurements and a more detailed separation of intrinsic and extrinsic magnetic contributions will be addressed in future work. Additionally, detailed microstructural characterization, including SEM imaging, will be performed to clarify the role of grain morphology and dopant distribution.

## Figures and Tables

**Table 1:** Compositional dependence of Curie temperature and substitution ratio of tetrahedral site, *z*/*x* in (Bi,Al)/YIG and (Bi,Ga)/YIG. (here “–” Indicates cases where the tetrahedral site occupancy ratio (*z*/*x*) could not be determined).

*x* (Al/Ga)	*T*_C_ for (Bi,Al)/YIG) [K]	*z*/*x* for (Bi,Al)/YIG	*T*_C_ for (Bi,Ga)/YIG) [K]	*z*/*x* for (Bi,Ga)/YIG
0	591	0	591	0
0.5	540.7	0.801	540.6	0.799
1.0	474.5	0.617	497.6	–
1.5	424.4	0.690	429.6	0.767
	405.5	0.550	468.7	–
1.7	454.4	–	502.9	–
	402.4	0.699	433.2	–
1.9	407.8	–	332.2	0.470
	403.3	–	389.2	–
			375.5	0.659
2.0	392.5	–	350.9	0.582
	395.6	–		
2.1	406.6	–	328.5	0.543
2.3	400.7	–	335.0	–
	399.1	–		

## Data Availability

The original contributions presented in this study are included in the article. Further inquiries can be directed to the corresponding author.
